# Clinical outcomes and survival rates of a uncemented modular revision stem system in hip arthroplasty: a 10-year single-institution study on a frail population

**DOI:** 10.1007/s00402-024-05483-3

**Published:** 2024-08-10

**Authors:** Daniele De Meo, Paolo Martini, Beatrice Perciballi, Giovanni Guarascio, Matteo Vacca, Gianluca Cera, Stefano Gumina, Ciro Villani

**Affiliations:** 1 Department of Anatomical, Histological, Forensic Medicine and Orthopaedics Sciences, Sapienza University of Rome, Piazzale Aldo Moro, 3, Rome, 00161 Italy; 2 Department of General Surgery, Plastic Surgery and Orthopaedics, Policlinico Umberto I University Hospital, Rome, Italy; 3 Emergency Department, Policlinico Umberto I University Hospital, Rome, Italy; 4 Istituto Clinico Ortopedico Traumatologico (ICOT), Latina, Italy

**Keywords:** Revision total hip arthroplasty, Uncemented, Stem, Periprosthetic joint infection, Aseptic loosening, Periprosthetic fracture, Revision surgery, Complication, Modular hip stem

## Abstract

**Introduction:**

The increasing prevalence of primary hip arthroplasty has led to a parallel rise in revision cases. Femoral revision often entails compromised bone integrity, requiring consideration of various solutions for optimal reconstructive options. Despite technological advancements, there is limited evidence on the clinical outcomes of the latest modular revision stems. This study aimed to evaluate the clinical outcomes and survival rates of next generation uncemented modular revision stem in patients undergoing hip revision surgery.

**Materials and methods:**

This retrospective single-center study assessed the survival and failure causes of a specific uncemented modular stem in 48 patients undergoing hip revision surgery between 2012 and 2022. Data included preoperative parameters, surgical details, and postoperative outcomes measured through clinical and radiographic assessments. Forty-eight patients (25 males, 23 females; mean age 72 years) were included, with a mean Charlson Comorbidity Index of 5. Preoperative diagnoses varied, with periprosthetic joint infection (PJI) being the most common (45.8%), followed by periprosthetic fractures (27.1%). Partial revisions occurred in 60.4%, total revisions in 39.6%. According to Paprosky classification of femoral bone loss, type II and III were the most represented, respectively 35.4% and 50%.

**Results:**

At a mean follow-up of 4.6 years, stem survival was 92.5%. Complications (20%) included dislocation, PJI, fracture, and loosening; the overall reoperation rate was 12.5%. The SF-12 physical score was 43.6, while the mental score was 51.1. The HOOS score was 71.8, and the HHS score was 71.4. Radiographic analysis identified nonprogressive osteolysis in 15.1% of patients.

**Conclusions:**

This study on this uncemented modular revision stem demonstrated favorable outcomes in an elder fragile population with moderate to severe femoral bone loss. The implant’s modularity provides versatility in addressing various defects, without any implant breakage observed during the study period. Literature comparison highlighted similar outcomes despite sample size differences. The promising results warrant continued investigation into the long-term survivorship of this modular stem system.

## Introduction

Along with the increase in primary hip arthroplasty, the number of revisions is growing, with a projected 43–70% increase expected by 2030 [1, 2]. Indications for revision vary based on patient demographics. Among patients younger than 55 years at the time of primary surgery, aseptic loosening is the most common indication. In contrast, individuals aged 84 years and older exhibit a higher incidence of dislocation, periprosthetic fracture, and periprosthetic joint infection (PJI). Wear-related complications or component malposition account for a smaller proportion (5%) [3].

Femoral revision is frequently complicated by bone loss or compromised integrity of the remaining bone stock. In preoperative planning, it is crucial to identify the location of femoral bone defects and classify them, facilitating improved reconstructive options for the patient. This becomes especially pertinent when stem removal becomes challenging or when significant bone loss is identified intraoperatively [4]. Multiple fixation options, such as cemented or uncemented components, and patient-specific implants, must be considered for femoral reconstruction in such challenging conditions [5].

Despite the continuous introduction of new technologies and designs, there remains a paucity of evidence in the literature regarding the clinical outcomes and survival rates of the latest generation revision stems. Therefore, the objective of this study was to assess the survival rate and potential causes of failure of an uncemented modular revision stem implanted in a consecutive series of frail patients undergoing revision hip arthroplasty over a 10-year period in a single institution. The study aims to comprehensively evaluate both clinical and radiographic outcomes.

## Materials and methods

### Study design and setting

A retrospective observational study was conducted on patients treated at a single istitution, Policlinico Umberto I University Hospital, who underwent hip revision surgery between January 1, 2012, and June 30, 2022. Informed consent regarding the surgery, as well as the collection and analysis of data, was obtained from all individual participants included in the study.

Patients undergoing both partial and complete revision hip surgery were included, specifically those in which the Arcos^®^ modular stem (Zimmer Biomet Inc., Warsaw, Indiana, USA) was utilized, with a minimum follow-up period of one year. Exclusion criteria encompassed incomplete hospitalization data, patients unavailable for follow-up, and systemic cancer with a pre-surgery estimated prognosis of less than 6 months.

All patients were operated on by the same surgical team. Routinely, preoperative evaluation and planning was performed on x-ray and CT scan. The modular cementless stem is usually chosen by the senior surgeon in all the cases in which the bone loss is not completely predictable, regardless of diagnosis (PJI, aseptic loosening, periprosthetic fracture, etc…). The Arcos^®^ modular stem is a cementless system featuring three proximal and five distal geometry options, providing various combinations and auxiliary fixation options. This extensive variability in combinations allows for the selection of an appropriate anchor system based on the patient’s bone defects. Standard preoperative antibiotic prophylaxis with cefazolin was administered, and in cases of periprosthetic joint infection (PJI), targeted therapy was initiated if a preoperative isolate was identified. Tranexamic acid was used, if not contraindicated, to reduce bleeding. A posterolateral surgical approach was employed for all revisions. Quadriceps and gluteus strengthening, and the assumption of the sitting position was allowed immediately. Weight-bearing was permitted based on general conditions, and in cases where a trochanteric osteotomy had been performed, complete weight loading was deferred according to the size of the osteotomy. Outpatient follow-up visits, including radiographic evaluations, were customized to each patient, typically following a schedule of visits at 1, 3, 6, and 12 months after surgery, followed by annual visits for the first five years.

Reviewing all electronic medical records, preoperative clinical parameters included anamnestic features and comorbidities, represented by the Charlson Comorbidity Index (CCI) [6]. Preoperative hemoglobin value and preoperative bone stock, according to the Paprosky classification of femoral bone loss, were also documented [7].

### Demographic data

48 patients were enrolled in the study, comprising 25 males and 23 females, with an average age of 72 ± 20 years. The CCI value was 5 ± 1.4, and the average BMI was 26.3 ± 5.8 kg/m². Forty-five patients had more than one comorbidity. Comorbidities are listed in Table 1. The pre-operative diagnoses were as follows: periprosthetic joint infection (PJI) in 22 patients (45.8%), periprosthetic fracture in 13 patients (27.1%), aseptic loosening in 10 patients (20.8%), painful prosthesis in 2 patients (4.2%), and breakage of the first implant prosthesis in one patient (2.1%). Femoral bone loss according to the Paprosky classification was distributed as follows: Paprosky I in 2 patients (4.2%), Paprosky II in 17 patients (35.4%), Paprosky IIIA in 18 patients (37.5%), Paprosky IIIB in 6 patients (12.5%), and Paprosky IV in 5 patients (10%). The average time to failure from the index procedure was 108 ± 86 months, and the mean hemoglobin (Hb) value was 12 ± 2.04 mg/dl [Table 2].

**Table 1:** Anamnestic data of the sample

Demographic		
Gender		
Male	25 (52%)	
Female	23 (48%)	
Age (years)	72 ± 12	
BMI (kg/m^2^)	26.3 ± 5.8	
Smoke	15 (31.3%)	
Drugs	2 (4.2%)	
Anemia	9 (18.8%)	
Diabetes	7 (14.6%)	
Tumors	11 (22.9%)	
Chronic renal failure	4 (8.3%)	
Heart failure	7 (14.6%)	
Previous myocardial infarction	3 (6.3%)	
COPD	7 (14.6%)	
Peripheral vascular disease	3 (6.3%)	
Cerebrovascular disease	2 (4.2%)	
Dementia	5 (10.4%)	
Hemiplegia	2 (4.2%)	
Peptic ulcer	2 (4.2%)	
CCI	5 ± 1.4	

BMI: body mass index; COPD: chronic obstructive pulmonary disease; CCI: Charlson comorbidity index

**Table 2:** Pre-operative diagnosis and paprosky class of the pre-operative radiographs

Pre-operative data	
Diagnosis	
Periprosthetic joint infection	22 (45.8%)
Periprosthetic fracture	13 (27,1%)
Aseptic loosening	10 (20.8%)
Painful prosthesis	2 (4.2%)
Stem breakage	1 (2.1%)
Paprosky classification of femoral bone loss	
I	2 (4.2%)
II	17 (35.4%)
IIIA	18 (37.5%)
IIIB	6 (12.5%)
IV	5 (10.4%)
Time to failure	108 ± 86 months
Hb pre-operative	12 ± 2,04 mg/dl

A partial revision, involving the prosthetic stem only, was performed in 29 patients (60.4%), while a total revision, which included the replacement of both the stem and the cup, was conducted in 19 patients (39.6%). In cases of total revision, the Zimmer Biomet G7^®^ Acetabular System uncemented cup was used in 18 patients, whereas the Zimmer Biomet Avantage ^®^ Acetabular System cemented cup was used in 1 patient. Extended trochanteric osteotomies (ETO) [8] were performed in 8 patients (16.7%). Internal fixation devices were utilized in 30 patients, including cerclage wires in 17 patients (35.4%), plates and screws in 2 patients (4.2%), and a combination of plates and cerclages in 11 patients (22.9%). No fixation device was used in 18 patients (37.5%). Notably, there were no intraoperative iatrogenic fractures in our sample. Bone grafts were employed in 5 patients (10.4%). Intraoperative data and modular stem characteristics in our sample are detailed in Table 3. The average length of stay was 14 ± 18 days, and blood transfusions were administered to 21 patients.

**Table 3:** Intraoperative data and modular stem characteristics

Intra-operative data	
Type of revision	
Partial revision (only stem)	29 (60.4%)
Total revision with	19 (39.6%)
-G7® Acetabular System	18
-Avantage ® Acetabular System	1
Fixation devices	
Plates and cerclages	11 (22.9%)
Cerclages	17 (35.4%)
Plates and screws	2 (4.2%)
None	18 (37.5%)
Proximal body types	
Cone	34 (70.8%)
Limestone	/
Broached	14 (29.2%)
Stem types	
STS Splined tapered	30 (62.5%)
Slotted	/
Bullet tip	16 (33.3%)
Interlocking	1 (2.1%)
ETO	1 (2.1%)
Couplings (proximal body + distal stem)	
Cone + STS Splined tapered	20 (41.7%)
Cone + Bullet- tip	12 (25%)
Broached + STS Splined tapered	10 (20.8%)
Broached + Bullet-tip	4 (8.3%)
Cone + Interlocking	1 (2.1%)
Cone + ETO	1 (2.1%)
Average length (proximal body + distal stem)	213 ± 27 mm
Sleeve	
-6 mm	12 (25%)
-3 mm	15 (31.2%)
+ 3 mm	7 (14.6%)
+ 6 mm	2 (4.2%)
0 mm	12 (25%)
Material couplings	
Ceramic-ceramic	28 (58.3%)
Dual mobility	20 (41.7%)
Additional procedures	
ETO	8 (16.7%)
Bone grafts	5 (10.4%)

STS: splined tapered stem; ETO: extended trochanteric osteotomy

### End-points and follow-up

At the follow-up visit, conducted after a minimum of 12 months, patients were interviewed regarding the clinical progression post-surgery and underwent both clinical and radiographic assessments. The primary outcome measured was the survival of the Arcos^®^ stems. Secondary outcomes included the complication rate, specifically periprosthetic joint infection or implant loosening, and postoperative functionality assessed through questionnaires. Clinical postoperative parameters were evaluated using the Hip Disability and Osteoarthritis Outcome Score (HOOS) [9], the Harris Hip Score (HHS) [10], and Short Form − 12 (SF-12) [11], recorded at the last follow-up.

Postoperative radiographic parameters encompassed signs of loosening, subsidence, or osteolysis within the Gruen zones [12], as observed in the radiograph performed at the last follow-up. Both preoperative and postoperative radiographs were reviewed by two residents in Orthopedics and Traumatology and validated by an expert surgeon. Any discrepancies in the evaluations were resolved by the expert surgeon.

### Statistical analysis

Statistical analysis was performed using R 4.2.2 software (R Foundation for Statistical Computing©, Vienna, Austria). All acquired values are presented as mean, standard deviation, and/or 95% confidence interval. The Kaplan-Meier analysis will estimate the percentage of implant survival.

## Results

### Clinical outcomes

At the last follow-up, 40 patients remained available. Eight patients (16.7%) died during the follow-up period due to causes unrelated to the surgery. With an average follow-up duration of 4.6 years, the survivorship of the stem was found to be 92.5% [see Table 4]. Kaplan-Meier curve is depicted in Table 5.

**Table 4:** Clinical and radiographic outcomes

Results	
Deaths	8 (16.7%)
Patients without complications	32 (80%)
Patients with complications	8 (20%)
Dislocation	4 (10%)
PJI	2 (5%)
Aseptic loosening	1 (2.5%)
Periprosthetic fracture	1 (2,5%)
Additional surgical procedures	5 (12.5%)
Stem revision	3 (7.5%)
Clinical results	
HOOS	71.8 ± 21.6 (18.8–98)
HHS	71.4 ± 23.6 (12.5–95)
SF-12 (Physical score)	43.6 ± 11.5 (17.6–57.7)
SF-12 (Mental score)	51.1 ± 10.9 (14.7–62.9)
Radiological results	
Osteolysis (classified by Gruen’s zones)	5 (12.5%)
Zone 1	1 (2.5%)
Zone 6	2 (5%)
Zones 1 + 2	1 (2.5%)
Zones 1 + 7	1 (2.5%)
Length of stay	14 ± 18 days
Follow-up	55 ± 35 months

PJI: periprosthetic joint infection; HOOS: hip disability and osteoarthritis outcome score; HHS: Harris Hip score; SF-12: short form – 12

**Table 5:**
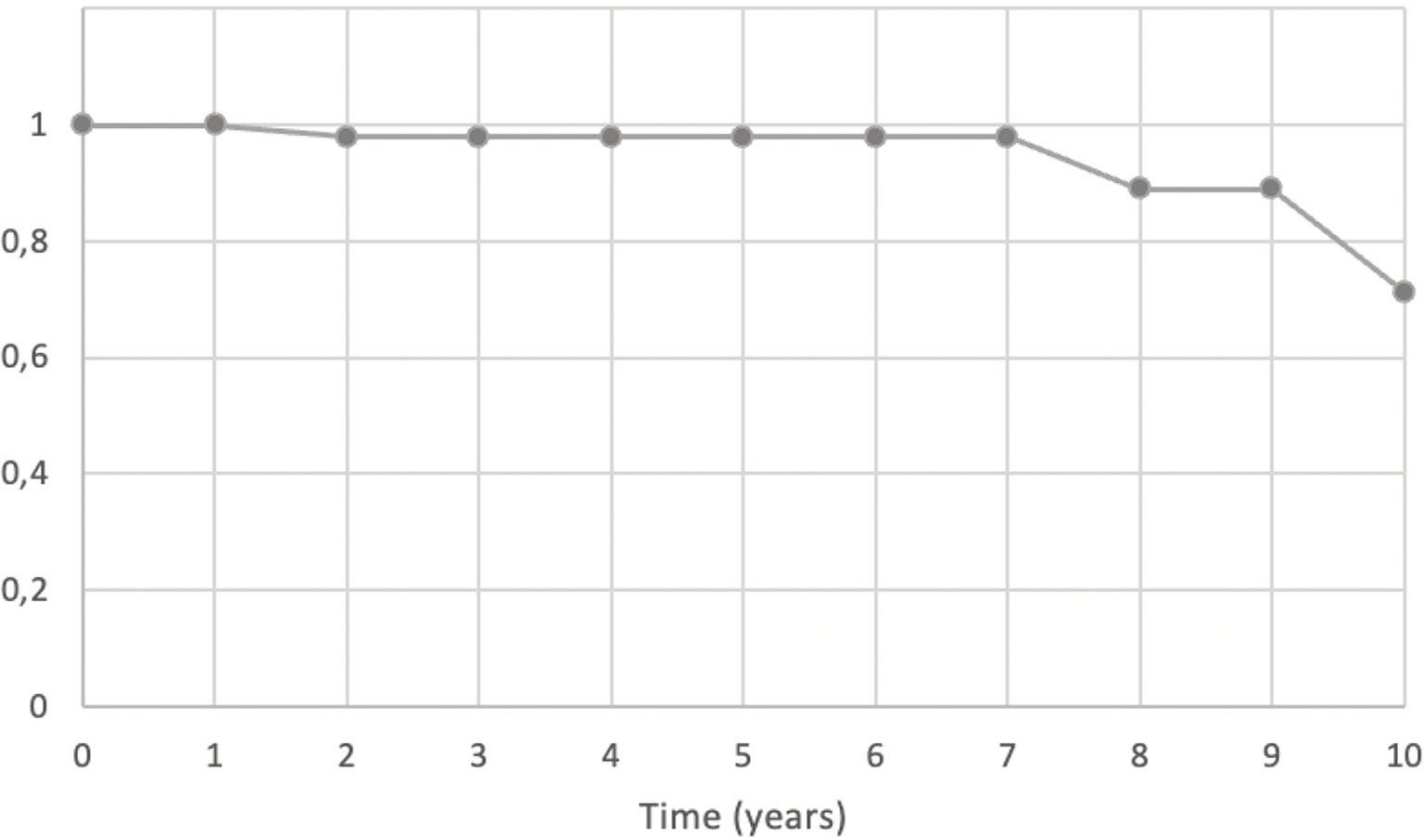
Kaplan-Meier survival curve

Among the remaining patients, eight individuals (20%) experienced major complications: four cases of dislocation (10%), two cases of periprosthetic joint infection (PJI) (5%), one case of periprosthetic fracture (2.5%), and one case of aseptic loosening (2.5%). For three patients with dislocation, conservative treatment with a reduction maneuver was successful without recurrence. However, five patients required additional surgical procedures (12.5%): those with chronic PJI underwent a two-stage exchange, the irreducible dislocation case was addressed with prosthetic head and sleeve substitution, the periprosthetic fracture was managed with open reduction and internal fixation (ORIF) without stem revision, and the loosening case underwent stem revision. Stem revision was performed in three cases (7.5%).

The average SF-12 physical score was 43.6 ± 11.5, while the average mental score was 51.1 ± 10.9. The mean HOOS score was 71.8 ± 21.6, and the average HHS score was 71.4 ± 23.6.

### Radiographical outcomes

Osteolysis was identified in 5 patients (12.5%), and its distribution was classified according to Gruen’s zones: zone 1 in one patient (2.5%), zone 6 in two patients (5%), zones 1 and 2 in one patient (2.5%), and zones 1 and 7 in one patient (2.5%). Importantly, none of the patients with osteolysis reported clinical symptoms or complications.

## Discussion

Revision prosthetic surgery carries a fivefold greater risk of complications compared to primary prosthetic surgery, with aseptic loosening (23.19%) and periprosthetic infection (22.13%) ranking as the primary causes of failure [13]. Our retrospective analysis demonstrated a survival rate of 92.5% for the Arcos^®^ stem over an average follow-up of 4 years and 6 months. This contrasts with an all-causes reintervention rate of 12.5% and a death rate of 16.7% due to non-orthopedic causes. A lone study in the literature exploring Arcos^®^ outcomes reported a survival rate of 96% [14]. Dyreborg et al. studied 116 patients with a mean follow-up of 4 years (0.5-6), with only 46 patients available for clinical examination during follow-up. Primary causes of revision in their study were aseptic loosening (69%), periprosthetic fracture (15%), and PJI (12%), while in our sample, the predominant causes were PJI (45.8%) and periprosthetic fracture (27.1%). The variation in survival scores is attributed to the difference in revision causes, with PJI being more prevalent in our study.

Implant breakage, a recognized complication in modular revision hip stems [15, 16], is often associated with inadequate proximal bone support [16–18]. Micromovements due to inadequate support result in wear and, consequently, stem fracture [19]. Factors increasing fracture risk include elevated BMI, small diameter stem, and over-extended trochanteric osteotomy [16]. Remarkably, this complication was not observed in our study. Literature reports only two case reports detailing catastrophic failures with the Zimmer Biomet Arcos^®^ Revision System. The first case described a specific fracture involving a splined tapered stem (STS) at the junction with a proximal conical body, occurring in a young patient with an elevated body mass index and proximal bone deficiency [20]. The second case reported screw failure in the Trochanteric Bolt and Claw Technique in a 74-year-old male patient who underwent revision of a previous hip resurfacing due to an intertrochanteric hip fracture with subtrochanteric extension [21]. The scarcity of literature on this type of complication underscores its unpredictable and multifactorial nature, making generalization inappropriate. The authors recommend meticulous effort in selecting the smallest length of the neck sleeve (or head) and the shorter height of the proximal body of the modular construct when using a modular revision stem, to minimize leverage forces between modular interfaces.

Comparison with published work on complications associated with other stem types reveals similar data despite our smaller sample size (40 vs. 115 ± 82 patients) and equivalent follow-up periods (4.6 vs. 5.2 ± 1.1 years) [Table 6] [22–33].

**Table 6:** Comparison between different modular revision stem and the results of Arcos stem in the current study

Stem	Number of patients	Mean follow-up (years)	Rate of revision surgery	Rate of dislocation	Rate of infection	Rate of aseptic loosening
Zimmer Biomet Arcos	40	4,6	7,5%	10%	5%	2,5%
MRP Titan^25^	79	4	4%	5%	3%	3%
Wright Profemur-R^26^	49	6,2	6%	2%	2%	4%
Lima-Lto^27^	62	4,2	4,8%	5%	2%	0%
Lima MRS^28^	316	5	9.8%	1,6%	5,7%	2,2%
Link MP^29^	90	6	10%	19%	3%	2%
Stryker Restoration Modular^30^	122	4	8%	3%	2%	0%
Stryker Restoration Modular^31^	86	4,3	11,6%	2,3%	4,7%	0%
Stryker Restoration Modular^32^	161	5,9	14,9%	4,3%	6,2%	2%
Biomet Mallory/Head^32^	75	7	11%	5%	7%	3%
Total	1080	5,2	8,79%	5,24%	4%	1,80%

Moving on to analyze the clinical results of the submitted questionnaires, the physical component of SF-12 was lower than the values from general population data (50.8 ± 8.9) [34]. The mean value of the mental component of the score was slightly higher than the general population (50.0 ± 9.5). The HOOS values are similar to the ones recorded in aseptic revision reported in the literature [35], but higher than patients who underwent one-stage (68.88 ± 9.47) or two-stage (68.17 ± 8.28) septic revisions [36]. HHS scores at the final follow-up were lower than values in the literature with average postoperative scores ranging from 81 to 85.6 [14, 32, 37]. These clinical outcomes must be interpreted considering the older age of our sample, the high CCI mean value, indicating multiple comorbidities, and the presence of PJI as the main reason for revision. Moreover, during the interviews, we noted that most of the patients found difficulties in ordinary or extraordinary activities due to other orthopedic problems, such as arthrosis of other joints or spine-related problems (hernias and spondylolisthesis).

The preoperative radiographic study was based on Paprosky classification of femoral bone loss. In the literature, approximately 80% of patients undergoing hip prosthetic revision correspond to Paprosky’s type II [32], whereas in our study, this type was found in less than 40% of patients, while the majority of the patients accounted for more severe defects (type III and IV). The preoperative bone stock is a prognostic factor for the success of prosthetic revision [38, 39]. Radiographic data at the time of reevaluation in follow-up showed the presence of osteolysis in 15.1% of patients. However, the patients who showed complications at follow-up had no evidence of subsidence, loosening, or osteolysis at the last radiographic checkup. Pawar et al. [40] described subsidence in patients with Arcos revision (10% of patients with a mean value of 2.3 mm) in comparison to Reclaim (Depuy Synthes) (30% of patients with a mean value of 4.5 mm), with no relation to preoperative bone stock, BMI, stem length, ETO, and none of them underwent further surgical procedures.

The generalization of this study can be considered medium to low. Being a single-center study, the data analyzed were derived exclusively from surgeries performed at our institution; moreover, the population examined is very heterogeneous, both in terms of the causes that led to revision surgeries and the grade of bone defect present at the level of the femoral component. There are several limitations of the study: first of all, the retrospective nature of the study. Secondly, the relatively low number of cases compared to other studies and the heterogeneous reasons for revisions in which this stem was used. Anyway, those limitations must be considered in the light of the relatively recent introduction of this last-generation modular revision stem. In addition, this study included only one type of implant, with no comparison group. Therefore, we cannot draw any definitive conclusions on the success of the Arcos^®^ system compared with other systems.

## Conclusions

The modular uncemented revision stem investigated in this study, within a frail population with moderate to severe femoral bone loss, yielded promising results with an acceptable revision rate. Its wide modularity allows versatility in addressing various femoral defects. Further studies must be conducted to assess the long-term survivorship of the implant.

## Data Availability

The datasets generated and analyzed during the current study are available from the corresponding author on reasonable request.
